# Supplementary material for: An unexpected role of CLASP1 in radiation response and S-phase regulation of head and neck cancer cells
Source: PLoS One. 2025 Aug 6;20(8):e0329731. doi: 10.1371/journal.pone.0329731 (PMC12327678; doi:10.1371/journal.pone.0329731)
Supplement: S1 File — (DOCX) [file pone.0329731.s001.docx]

**Supporting information**

**An unexpected role of CLASP1 in radiation response and S-phase regulation of head and neck cancer cells**

S1 Methods

S1-5 Figures

S1-3 Tables

**Supplementary methods 1**

*Screen analysis*

The raw demultiplexed FASTQ files were uploaded in CRISPRAnalyzer (http://crispr-analyzer.dkfz.de/) [1]: a web-based interface for mapping of the sequences and for quantification of the sequencing reads. In short, CRISPRAnalyzer aligns the unique sgRNA sequences to the reference library using BOWTIE2 mapping. The sgRNAs with low read counts (<20) were retained as depletion of these sgRNAs could be functionally underrepresented. Read counts obtained by CRISPRAnalyzer were used for further downstream analysis. We used the DrugZ pipeline to identify treatment-gene interactions by calculation of a Z-score per gene [2]. Both replicates were analyzed separately and a gene-irradiation interaction was assumed when the DrugZ score was at least below 0. Genes with a p-value <.05 on both replicates were expected to have a significant reproducible effect, and therefore assigned as hit. Alternatively we composed a NormZ score, in which the log2 transformed confidence interval of the read count ratio per sgRNA between treatment (2Gy irradiation) vs control (CI_irr_) and T_0_ vs T_11_ (i.e. essential genes; CI_ess_) was estimated using a Poisson distribution, with a confidence level of 1%. A log2 transformed read count ratio of 0 means that there has no change in sgRNA counts occurred after exposure to the condition of interest (irradiation or time). In order to increase robustness, we chose the limit of the log2 99%-CI nearest to 0 (i.e. 99.5% confidence that the observed read count ratio is not equal to 1) for downstream analysis. If the confidence interval crosses 0, the value for further analyses was assigned as 0. We calculated a Z-score per sgRNA by dividing the limit of the log2 99%-CI nearest to 0 by the standard deviation of all sgRNAs. Our Z-score was calculated for both replicates and for all sgRNAs, and we composed a Z-score per replicate per gene by adding up all Z-scores of all the sgRNAs. Genes with a combined Z-score < -10 in both replicates were assigned as hit. The two hit (DrugZ and NormZ) lists were merged. Functional enrichment analysis was performed with ShinyGO 0.77, an online gene-set enrichment tool.[3]

*Establishment and functional analysis of knockout cell lines*

The UM-SCC-11B Cas9 cell line was used to produce knockout cell lines for validation of hits of the screen, PRKDC knock-out cell lines were established as a positive control for radiosensitizing effect [4]. In short, UM-SCC-11B Cas9 cells were transfected with a CRISPR RNA (crRNA) of the different targets (See Supplementary Table 2 for specific sequences) and trans-activating crRNA (Dharmacon, U-002000-05) using Dharmafect 1 transfection reagent (Dharmacon, T-2001). As a control for transfection toxicity Edit-R crRNA Non-targeting Control (Dharmacon, U-007501) was taken as negative control and a crRNA targeting PLK1 an essential gene for HNSCC [5]: Edit-R Human PLK1_2 crRNA (Dharmacon, CM-003290-02), as positive control. When the controls indicated a successful transfection, monoclonal cell populations were obtained by seeding cells at a limiting dilution (1 cell per well) in 96 flat bottom well culture plates (Greiner Bio-One, Cat. No. 655160). After cell culture and when cells could be passaged, Mutation status was confirmed by Sanger sequencing with BigDye™ Direct Cycle Sequencing Kit (Applied Biosystems, Cat. No.4458687) according to the protocol of the manufacturer. Sequences were analyzed with ICE v2 CRISPR Analysis Tool [6].

*Western blotting*

Proteins were lysed and extracted using RIPA buffer (Thermo Scientific; Cat. No. 89901) containing the HALT protease and phosphatase inhibitor mix (Thermo Scientific; Cat. No. 78446). The protein sample was further prepared by adding 4x Laemmli sample buffer (Bio-Rad, Cat. No. 1610747) containing β-mercaptoethanol. Proteins were separated on 4-20% Mini-PROTEAN® TGX™ Precast Gels (Bio-Rad, Cat. No. 4561094), according to the protocol of the manufacturer. Proteins were transferred to an Immobilon-FL PVDF membrane (Millipore / Merck, IPFL00010), and blocked using Odyssey blocking buffer (LI-COR Biosciences, 927-70001). After first and second antibody incubations, signals were detected using the Odyssey® CLx Imaging System (Li-COR). The following antibodies were used for western blot analyses: Cas9 (7A9-3A3) (Cell Signaling; Cat. No. 14697), CLASP1 (Santa Cruz; Cat. No. sc-390159), DNA Ligase IV (Cell Signaling; Cat. No. 14649), a-Tubulin (B-7) (Santa Cruz; Cat. No. SC-5286), RNF8 (B2) (Santa Cruz; Cat. No. sc-271462), beta-Actin (13E5) (Cell Signaling; Cat. No. 4970) and DNA-PK (BD Biosciences; Cat. No. 556456) (Supplementary table 3).

*Clonogenic assay*

Cells were plated at an optimal density of 750 cells per T-25 culture flask (Greiner Bio-One, Cat. No. 690175), to allow exponential growth during the time of the experiment but with separate colonies. After 24 hours, the cells were treated with γ-radiation with a dose ranging from 0-4 Gray (Gy) on a ^60^Co source (Gammacell 220; MDS Nordion, Ontario, Canada), at room-temperature. At day 14, cells were fixed with formalin and stained with 0.5% crystal violet, and colonies >50 cells were manually counted by one observer.

*Etoposide IC50 determination*

Cells were plated in a 96-wells plate in a concentration of 2000 cells per well. After 24 hours of incubation, an eighteen step serial dilution (0.0008-100 µM) was made with etoposide (Selleckchem, S1225) and added to the cells. When a confluency of 80-90% was reached, Cell Titer Blue (CTB) (Promega, G8081) was added and incubated for 3 hours to monitor cell viability. Fluorescence measurements were performed on a Glomax explorer system (Promega, GM3500 and results were analyzed and visualized using Graphpad Prism 8.2.1. This program was also used to perform an unpaired t-test to compare the knockout cell lines to the parental cell line.

**Supplementary Figure 1, Genome wide CRISPR-cas9 knock-out screen control experiments in the HNSCC cellline UM-SCC-11B**

**A**

**
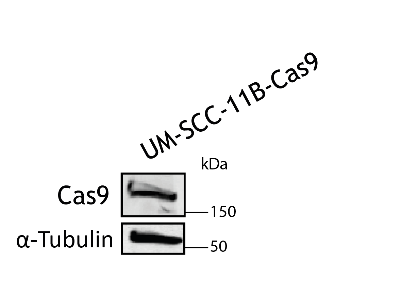
**

**B.**

**
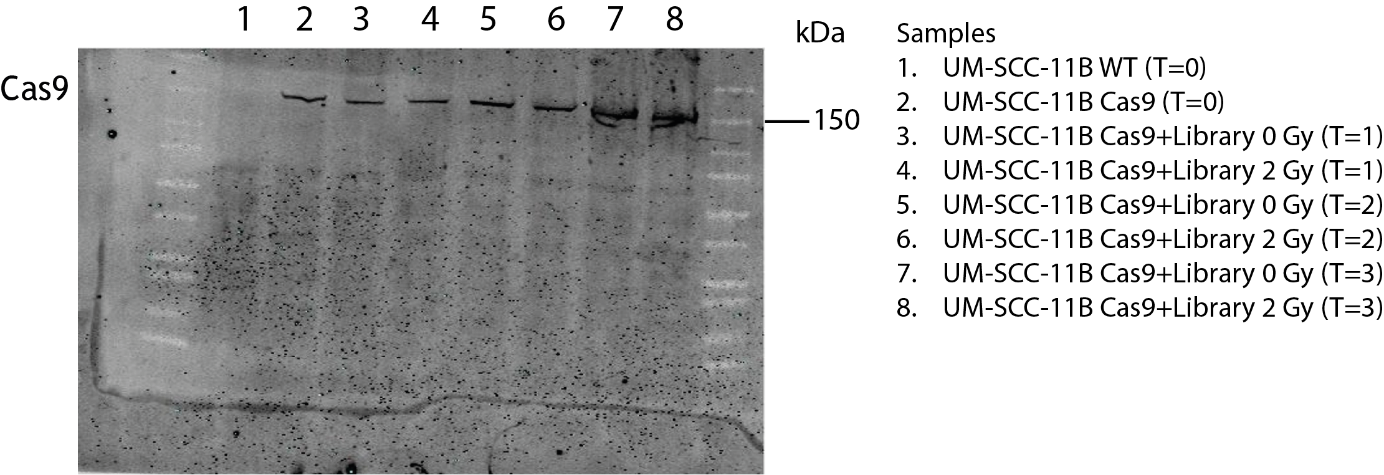
**

**C.**

**
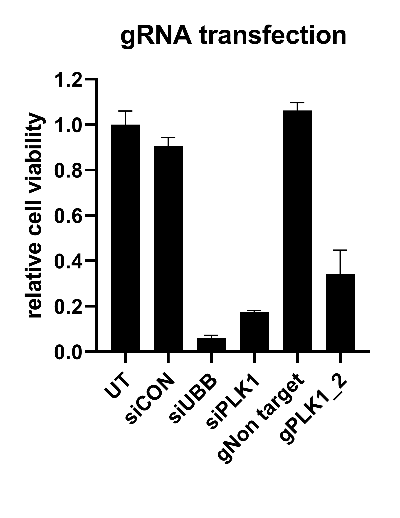
**

**D.
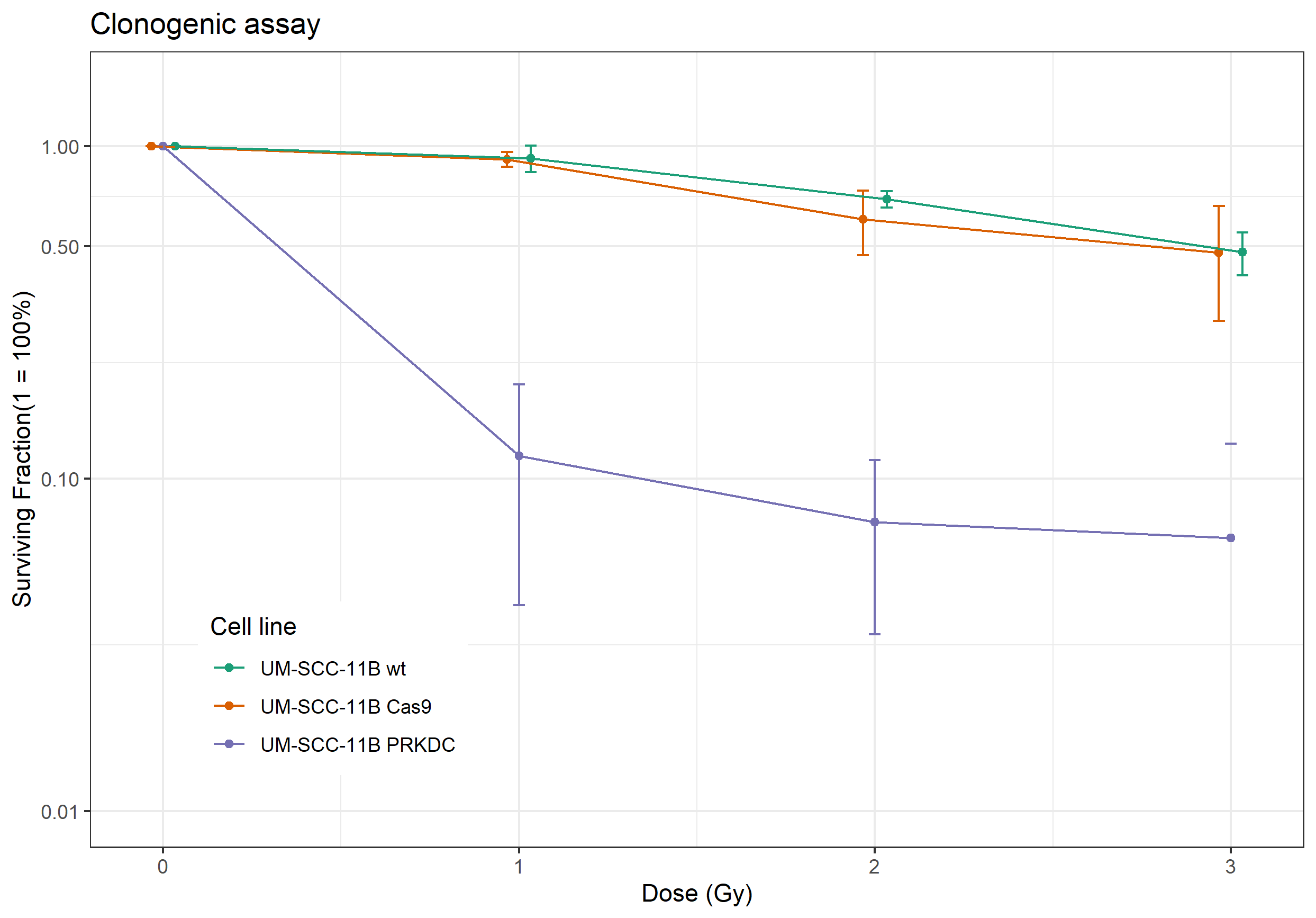
**

**Supplementary Figure 1: A)** UM-SCC-11B-Cas9 showed expression of Cas9 on Western blot analysis after lentiviral transduction with Cas9 expression vector conferring blasticidin resistance. **B)** Showing Western Blot analysis of Cas9 expression of our cell line during the CRISPR-Cas9 knock-out screen. **C)** Functional analysis of UM-SCC-11B-Cas9 showed comparable decrease of cell viability using a gRNA for a known lethal gene in HNSCC (*PLK1*) compared to conventional knock-down experiments using a siRNA for *PLK1*. **D)** Clonogenic assay of UM-SCC-11B parental, stable Cas9 expressing UM-SCC-11B and UM-SCC-11B PRKDC knock-out cell line (radiosensitizing control). Cells were plated at optimal density and after 24 hours treated with 0,1,2 or 3 Gy γ-radiation. At day 14 cells were fixed with formalin and stained with crystalviolet, colonies >50 cells were counted manually.

**Supplementary** F**igure** **2 Correlation of biological replicates of the CRISPR-Cas9 knock-out screen**


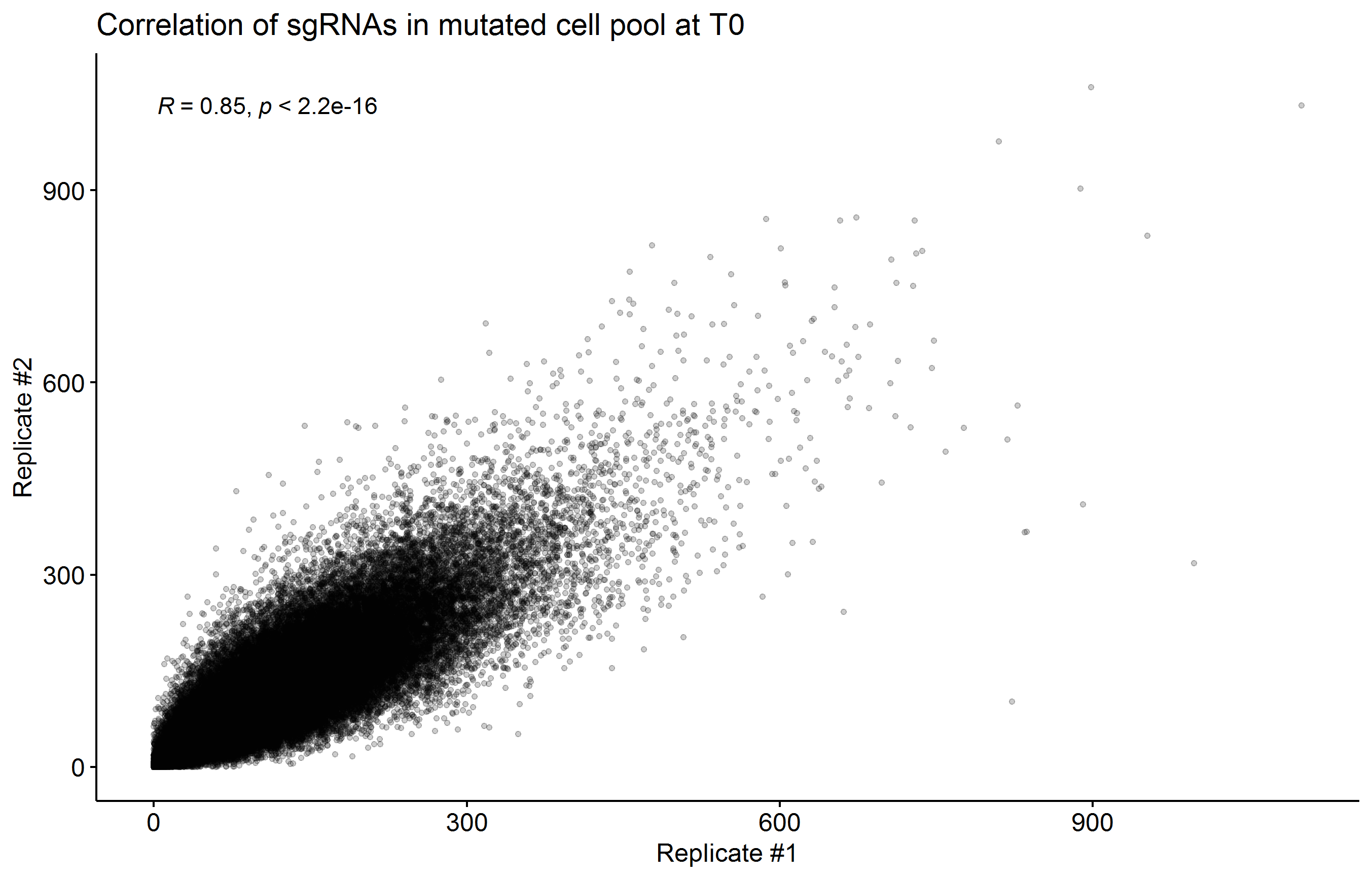


**Supplementary Figure 2:** The respective data from both replicates. Correlation of sgRNA reads in the mutated cell pool at T0 of both biological replicates show a good correlation (R=0.85).

**Supplementary Figure 3 Representation of core essential genes in our CRISPR-Cas9 screen, with and without irradiation**


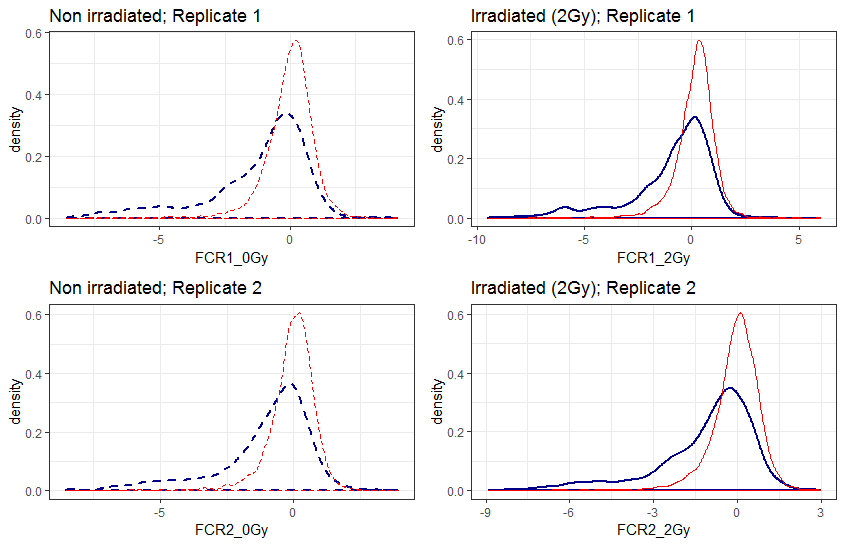


**Supplementary Figure 3:** Representative data from both replicates. The fold change distribution of all gRNA targeting essential genes (blue) is shifted relative to the fold change distribution of all gRNA targeting nonessential genes (red).

**Supplementary Figure 4 Electopherograms of clonally selected UM-SCC-11B Cas9 with *PRKDC*, *RNF8*, *LIG4* and *CLASP1* knocked-out.**

**
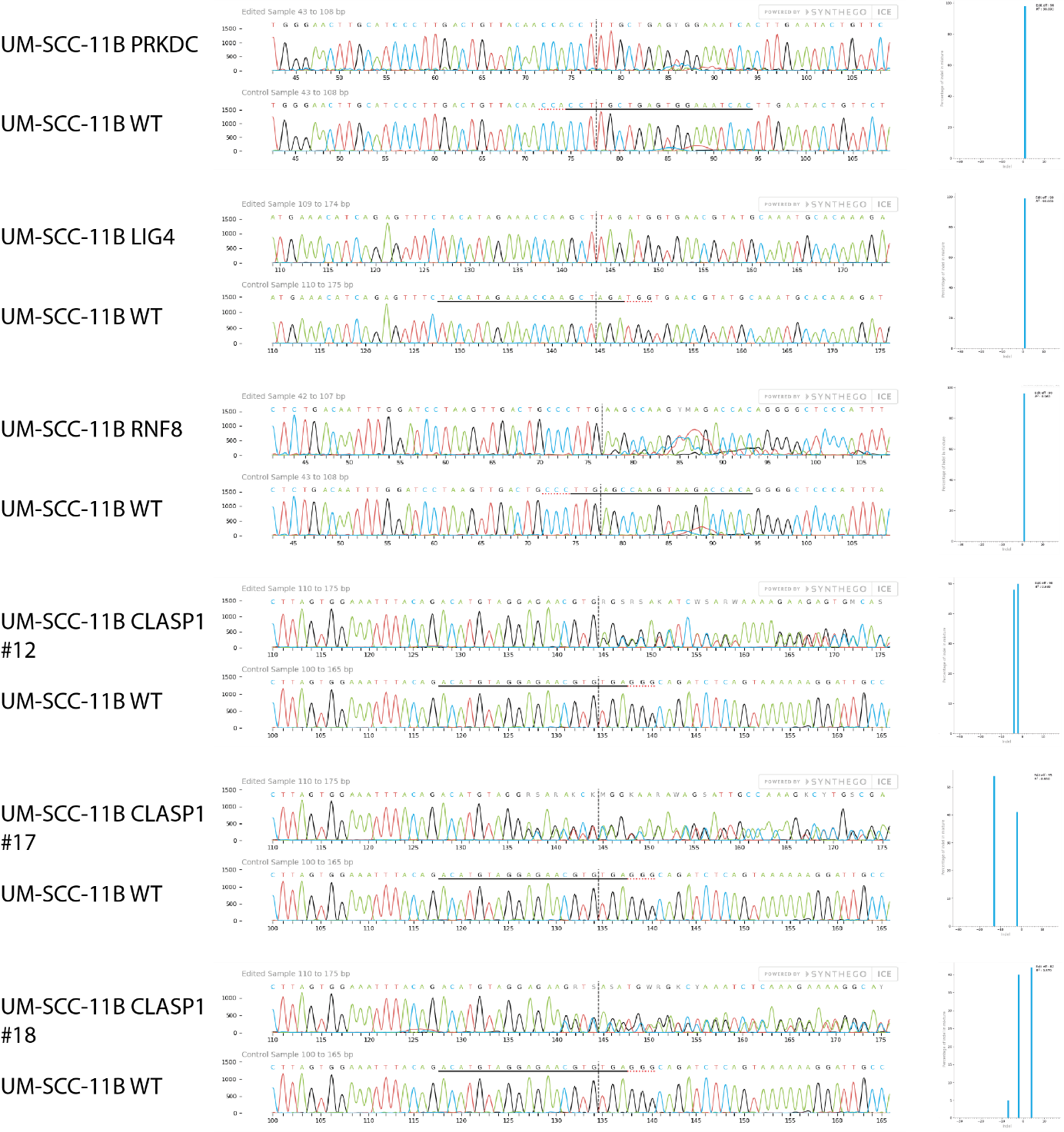
**

**Supplementary Figure 4:** Electopherograms of clonally selected UM-SCC-11B Cas9 with *PRKDC*, *RNF8*, *LIG4* and *CLASP1* knocked-out. After limiting dilution and clonal outgrowth, clones were sequenced and for the respective genes and analyzed by the ICE v2 CRISPR Analysis Tool[6].

**Supplementary Figure 5 Loss of CLASP1 sensitizes HNSCC cell line to the radiomimetic drug etoposide**

**A)**

**B)**

|  | UM-SCC-11B Cas9 | PRKDC | LIG4#1 (4) | CLASP1#1 (12) | CLASP1#1 (17) | CLASP1#1 (18) |
| --- | --- | --- | --- | --- | --- | --- |
| IC50 | 0,87 | 0,04 | 0,01 | 0,30 | 0,39 | 0,41 |

**Supplementary Figure 5:** IC50 determinations of knockout cell lines using the radiomimetic drug etoposide. Experiments are executed in three separate experiments with triplicates and the error bars represent standard deviation of nine values. **A**) IC50 of CLASP1 knockout cell lines. **B**) Table of the IC50 values of the different cell lines.

**Supplementary Table 1**

| **Name** | **Sequence** | **Used for** |
| --- | --- | --- |
| CRISPRSCREEN_PCR1_Fw | AGGGCCTATTTCCCATGATTCCTT | TKO Outer Fw |
| CRISPRSCREEN_PCR1_Rv | TCAAAAAAGCACCGACTCGG | TKO Outer Rv |
| CRISPRSCREEN_PCR2_Fw | AATGATACGGCGACCACCGAGATCTACAC-(8nt i5 index sequence)-ACACTCTTTCCCTACACGACGCTCTTCCGATCTTGTGGAAGGACGAGGACCG | TKO Fw i5 index 1 |
| CRISPRSCREEN_PCR2_Rv | CAAGCAGAAGACGGCATACGAGAT-(8nt i7 index sequence)-GTGACTGGAGTTCAGACGTGTGCTCTTCCGATCTATTTTAACTTGCTATTTCTAGCTCTAAAAC | TKO Rv i7 index primers |

**Supplementary Table 2**

| **Username** | **Gene of Interest** | **gene ID** | **crRNA sequence** | **Target exon** | **Genomic Location + PAM** | **Supplier** | **CAT.no** |
| --- | --- | --- | --- | --- | --- | --- | --- |
| PLK1 #2 | PLK1 | 5347 | GATCTCGGACGCGGACACCA | exon 1 | hg38-chr16: 23679139-23679161 AGG | Dharmacon / Horizon Discovery | CM-003290-02 |
| PRKDC #4 | PRKDC | 5591 | GTGATTTCCACTCAGCAAGG | exon 65 | hg38-chr8: 47821684-47821706 TGG | Dharmacon / Horizon Discovery | CM-005030-05 |
| CLASP1 #1 | CLASP1 | 23332 | ACATGTAGGAGAACGTGTGA | exon 7 | hg38-chr2: 121515699-121515721 GGG | Dharmacon / Horizon Discovery | CM-006831-01 |
| LIG4 #1 | DNA Ligase IV | 3981 | TACATAGAAACCAAGCTAGA | exon 3 | hg38-chr13: 108210442-108210464 TGG | Dharmacon / Horizon Discovery | CM-004254-01 |
| RNF8 #1 | RNF8 | 9025 | TGTGGTCTTACTTGGCTCAA | exon 3 | hg38-chr6: 37368869-37368891 GGG | Dharmacon / Horizon Discovery | CM-006900-01 |
| Non Targeting control #1 |  |  |  |  |  | Dharmacon / Horizon Discovery | U-007501 |
| Tracr RNA |  |  |  |  |  | Dharmacon / Horizon Discovery | U-002000-05 |

**Supplementary Table 3**

| **Primary antibodies** |  |  |  |  |
| --- | --- | --- | --- | --- |
| **Protein** | **leverancier** | **cat no.** | **kDa** | **RRID** |
| Cas9 (7A9-3A3) | cell signaling | 14697 | 160 | AB_2750916 |
| DNA Ligase IV | cell signaling | 14649 | 100 | AB_2750871 |
| CLASP1 (D-8) | Santa Cruz | sc-390159 |  | Na |
| a-Tubulin (B-7) | Santa Cruz | sc-5286 | 55 | AB_628411 |
| RNF8 (B2) | Santa Cruz | sc-271462 | 56 | AB_10648902 |
| beta-Actin (13E5) | cell signaling | 4970 | 45 | AB_2223172 |
| Beta-actin Clone AC-15 | Sigma | A5441 | 42 | AB_476744 |
| anti-Actin clone C4 | Millipore | MAB1501R | 42 | AB_2223041 |
| DNA-PK | BD Biosciences | 556456 | 350 | AB_396424 |
| phospho-Histone H2A.X (Ser139) clone JBW301 | Millipore | 650806 |  | AB_2564362 |
| **Secondary antibodies** |  |  |  |  |
| **Name** | **leverancier** | **cat no.** |  | **RRID** |
| anti-mouse Irdye® 680RD | Li-Cor | 926-68070 |  | AB_10956588 |
| anti-rabbit IRDye® 680RD | Li-Cor | 926-68071 |  | AB_10956166 |
| anti-mouse IRDye®800CW | Li-Cor | 926-32210 |  | AB_621842 |
| anti-rabbit IRDye®800CW | Li-Cor | 926-32211 |  | AB_621843 |
| Rat-anti-BrdU | Novus Biologicals | NB500-169 |  | AB_10002608 |
| mouse-anti-BrdU | BD Biosciences | 347580 |  | AB_400326 |
| goat-anti-mouse Alexa 488 | Life technologies | A28175 |  | AB_2536161 |
| goat-anti-rat Alexa 594 | Life technologies | A-11007 |  | AB_10561522 |

**References**

1. Winter J, Schwering M, Pelz O, Rauscher B, Zhan T, Heigwer F, et al. CRISPRAnalyzeR: Interactive analysis, annotation and documentation of pooled CRISPR screens. bioRxiv. 2017:109967. doi: 10.1101/109967.

2. Colic M, Wang G, Zimmermann M, Mascall K, McLaughlin M, Bertolet L, et al. Identifying chemogenetic interactions from CRISPR screens with drugZ. Genome Med. 2019;11(1):52. Epub 2019/08/24. doi: 10.1186/s13073-019-0665-3. PubMed PMID: 31439014; PubMed Central PMCID: PMCPMC6706933.

3. Ge SX, Jung D, Yao R. ShinyGO: a graphical gene-set enrichment tool for animals and plants. Bioinformatics. 2020;36(8):2628-9. doi: 10.1093/bioinformatics/btz931. PubMed PMID: 31882993; PubMed Central PMCID: PMCPMC7178415.

4. Azad A, Jackson S, Cullinane C, Natoli A, Neilsen PM, Callen DF, et al. Inhibition of DNA-dependent protein kinase induces accelerated senescence in irradiated human cancer cells. Mol Cancer Res. 2011;9(12):1696-707. Epub 2011/10/20. doi: 10.1158/1541-7786.MCR-11-0312. PubMed PMID: 22009179.

5. de Boer DV, Martens-de Kemp SR, Buijze M, Stigter-van Walsum M, Bloemena E, Dietrich R, et al. Targeting PLK1 as a novel chemopreventive approach to eradicate preneoplastic mucosal changes in the head and neck. Oncotarget. 2017;8(58):97928-40. Epub 2017/12/13. doi: 10.18632/oncotarget.17880. PubMed PMID: 29228663; PubMed Central PMCID: PMCPMC5716703.

6. Conant D, Hsiau T, Rossi N, Oki J, Maures T, Waite K, et al. Inference of CRISPR Edits from Sanger Trace Data. CRISPR J. 2022;5(1):123-30. Epub 20220202. doi: 10.1089/crispr.2021.0113. PubMed PMID: 35119294.
