# Supplementary material for: An unexpected role of CLASP1 in radiation response and S-phase regulation of head and neck cancer cells
Source: PLoS One. 2025 Aug 6;20(8):e0329731. doi: 10.1371/journal.pone.0329731 (PMC12327678; doi:10.1371/journal.pone.0329731)
Supplement: S2 File — (PDF) [file pone.0329731.s002.pdf]

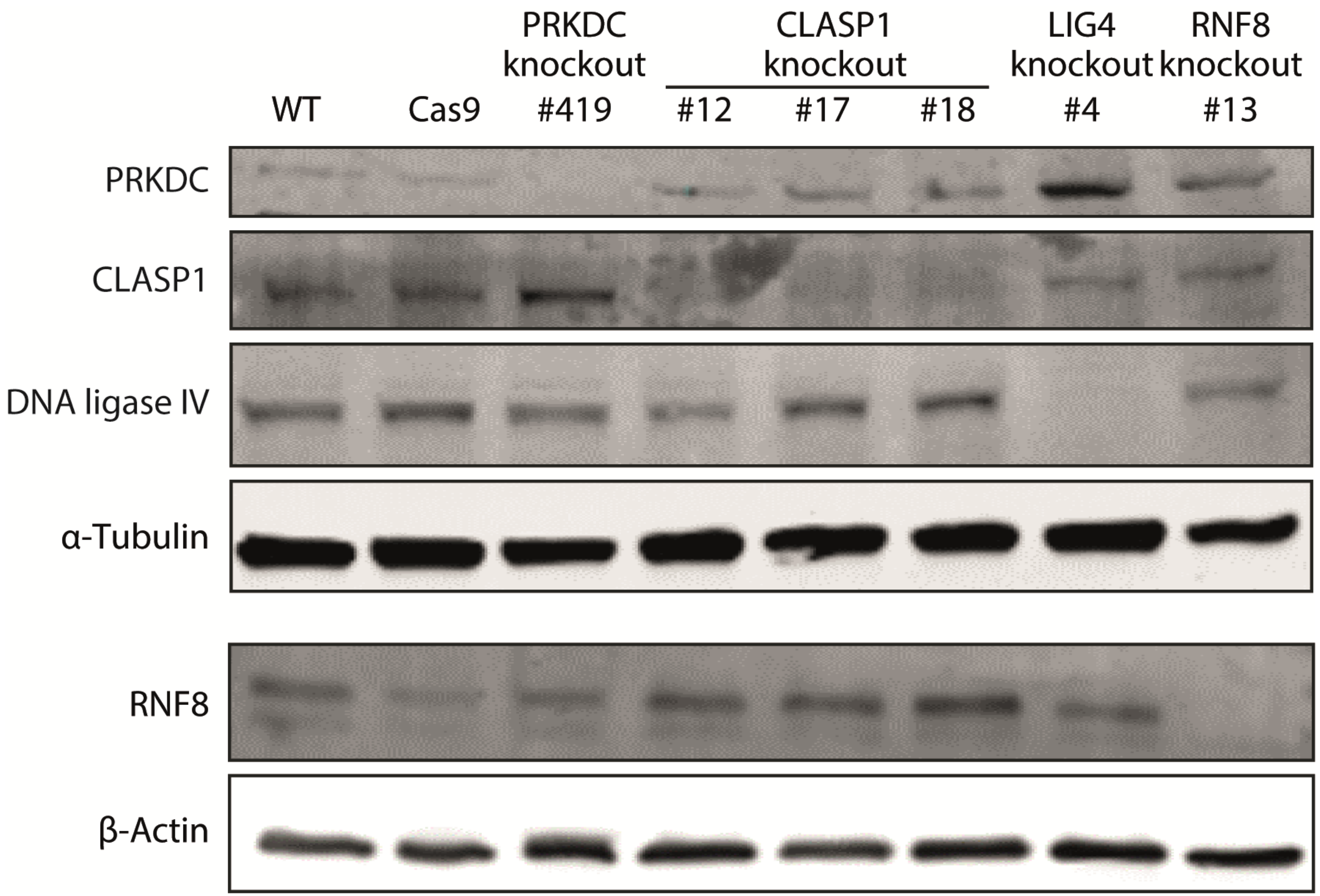

PRKDC knockout
CLASP1 knockout
LIG4 knockout
RNF8 knockout
WT
Cas9
#419
#12
#17
#18
#4
#13
PRKDC
CLASP1
DNA ligase IV
α-Tubulin
RNF8
β-Actin

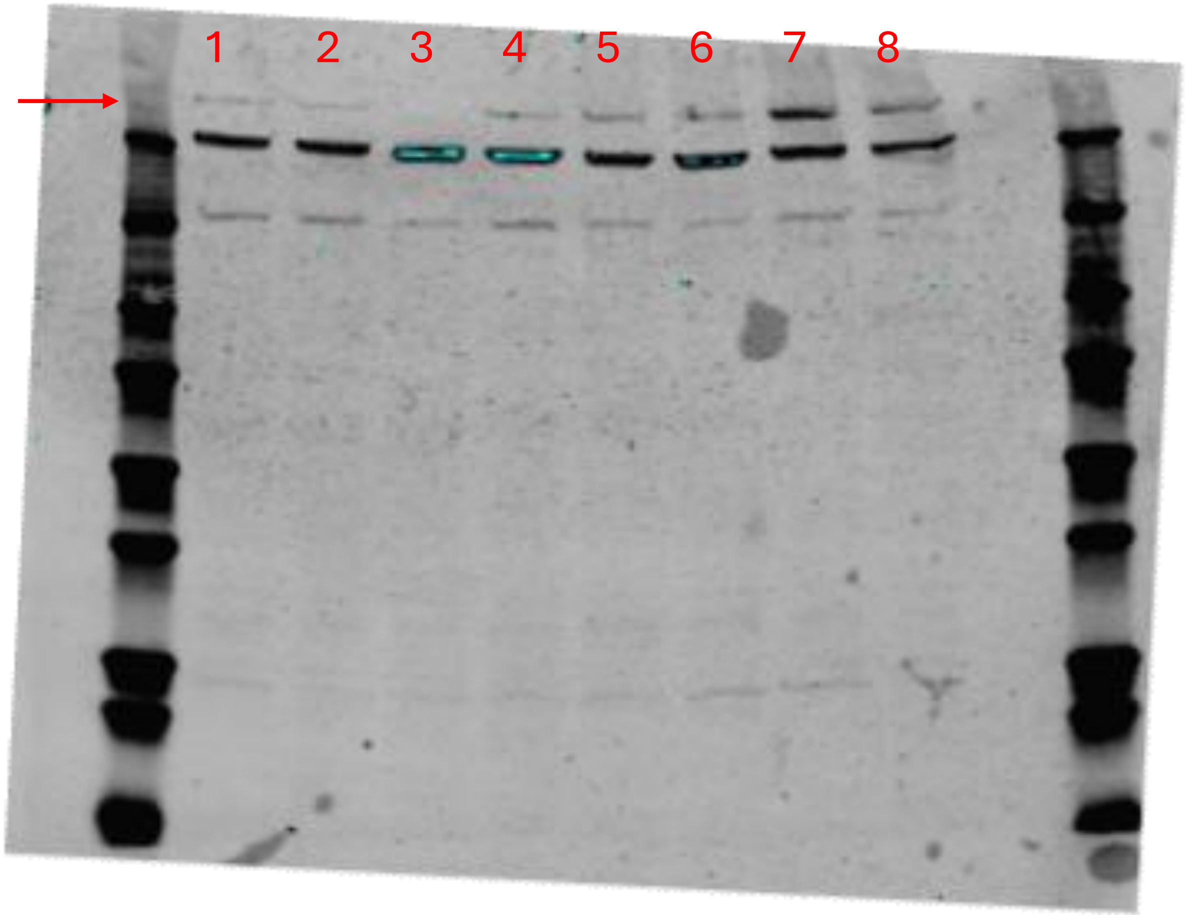

1. UM-SCC-11B-WT
2. UM-SCC-11B-Cas9
3. UM-SCC-11B-PRKDC
4. UM-SCC-11B-CLASP1 (12)
5. UM-SCC-11B-CLASP1 (17)
6. UM-SCC-11B-CLASP1 (18)
7. UM-SCC-11B-LIG4 (4)
8. UM-SCC-11B-RNF8 (13)

Western blot analysis shows the expression of *PRKDC* in the parental cell line, the Cas9 expressing cell line and the different knock-out cell lines.

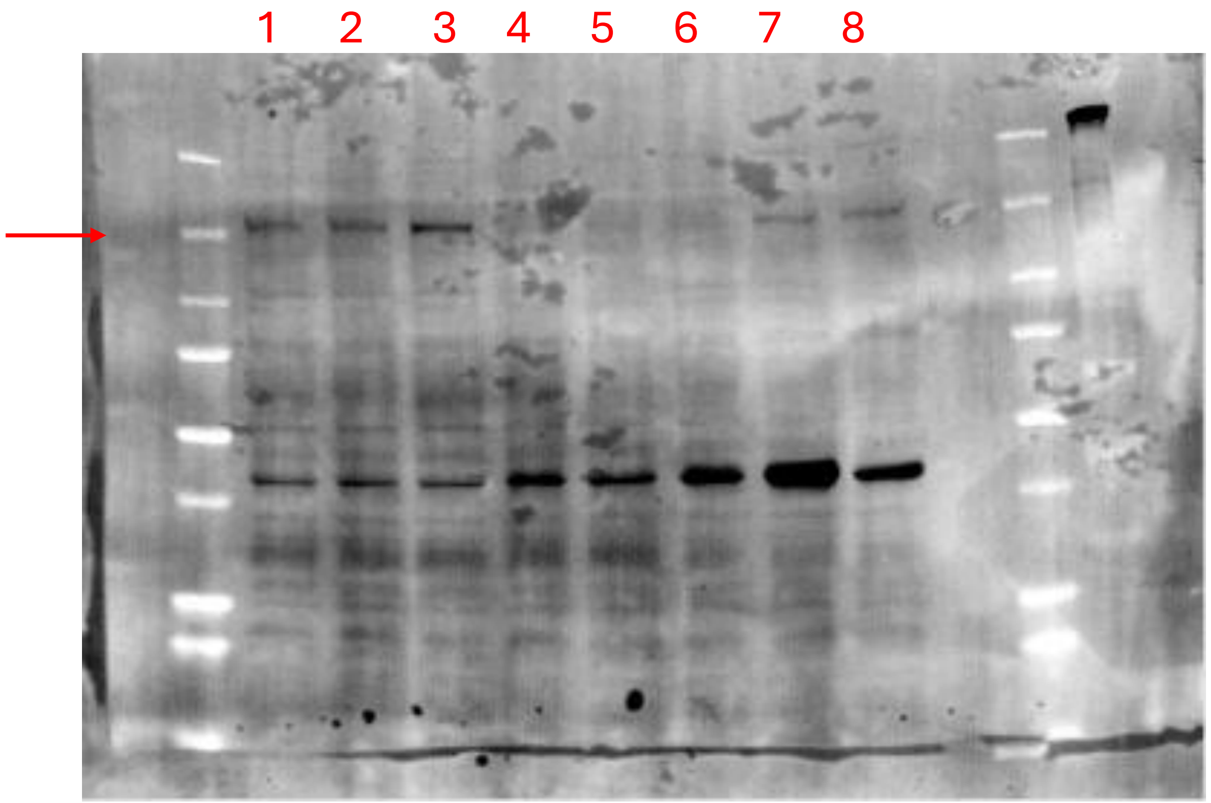

1. UM-SCC-11B-WT
2. UM-SCC-11B-Cas9
3. UM-SCC-11B-PRKDC
4. UM-SCC-11B-CLASP1 (12)
5. UM-SCC-11B-CLASP1 (17)
6. UM-SCC-11B-CLASP1 (18)
7. UM-SCC-11B-LIG4 (4)
8. UM-SCC-11B-RNF8 (13)

Western blot analysis shows the expression of *CLASP1* in the parental cell line, the Cas9 expressing cell line and the different knock-out cell lines.

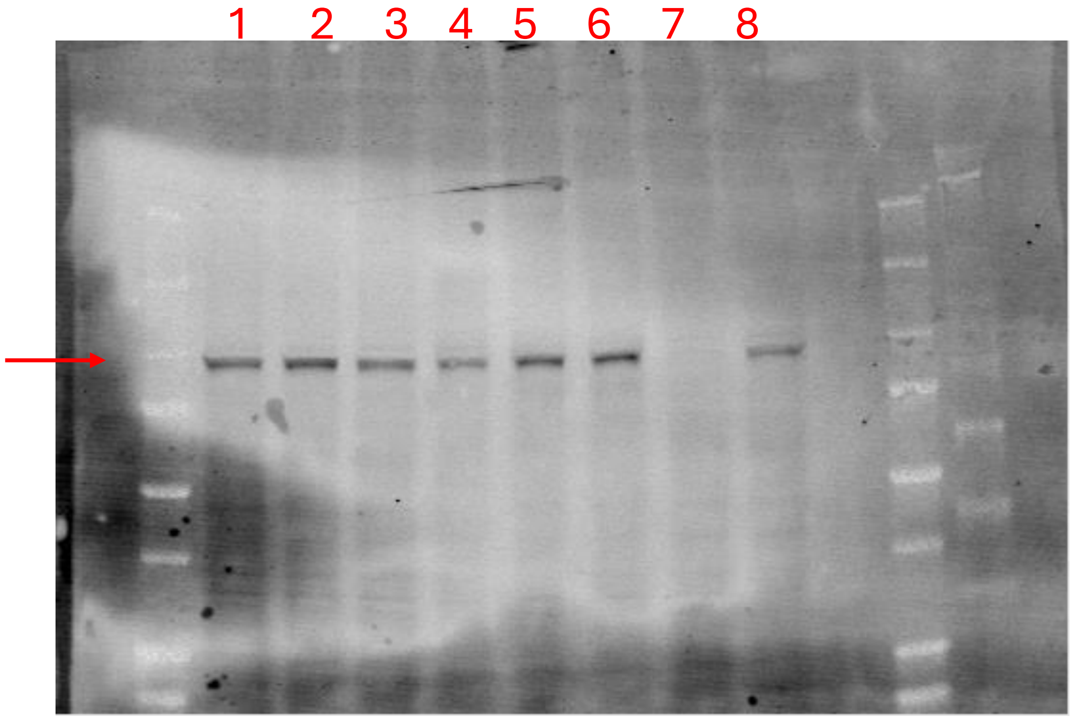

1. UM-SCC-11B-WT
2. UM-SCC-11B-Cas9
3. UM-SCC-11B-PRKDC
4. UM-SCC-11B-CLASP1 (12)
5. UM-SCC-11B-CLASP1 (17)
6. UM-SCC-11B-CLASP1 (18)
7. UM-SCC-11B-LIG4 (4)
8. UM-SCC-11B-RNF8 (13)

Western blot analysis shows the expression of *LIG4* in the parental cell line, the Cas9 expressing cell line and the different knock-out cell lines.

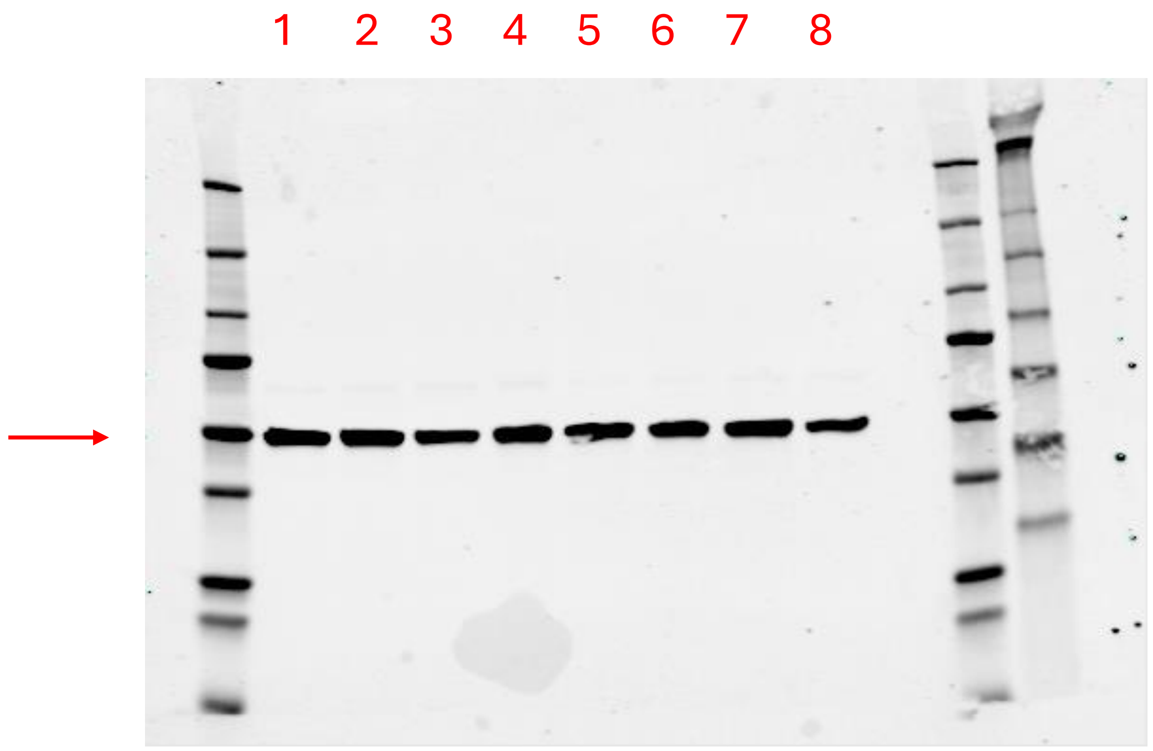

1. UM-SCC-11B-WT
2. UM-SCC-11B-Cas9
3. UM-SCC-11B-PRKDC
4. UM-SCC-11B-CLASP1 (12)
5. UM-SCC-11B-CLASP1 (17)
6. UM-SCC-11B-CLASP1 (18)
7. UM-SCC-11B-LIG4 (4)
8. UM-SCC-11B-RNF8 (13)

Western blot analysis shows the expression of *a-Tubulin* in the parental cell line, the Cas9 expressing cell line and the different knock-out cell lines.

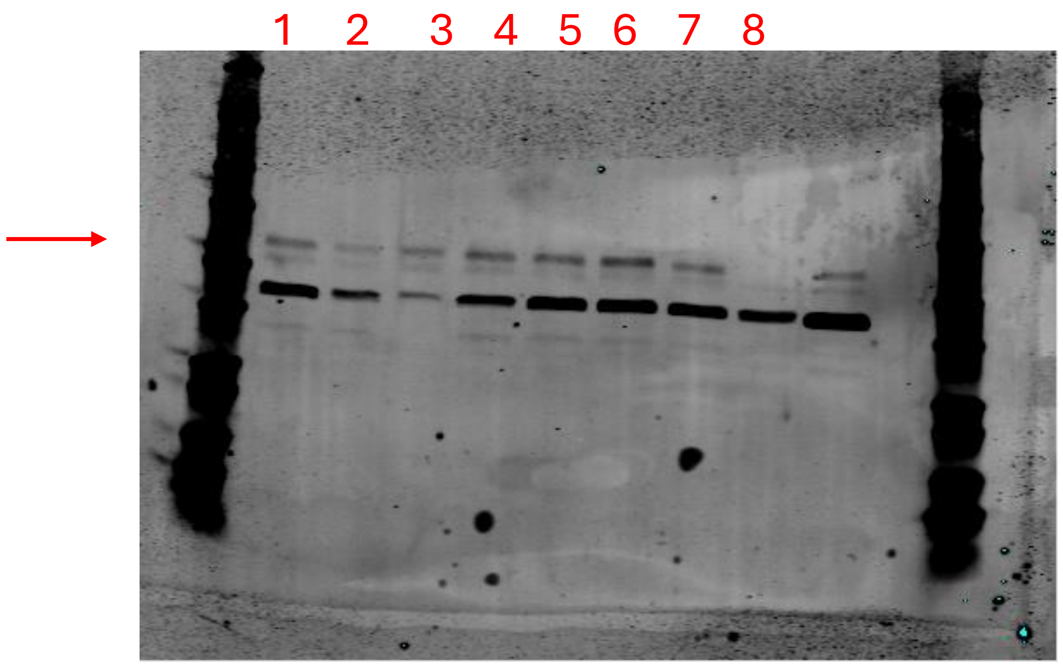

1. UM-SCC-11B-WT
2. UM-SCC-11B-Cas9
3. UM-SCC-11B-PRKDC
4. UM-SCC-11B-CLASP1 (12)
5. UM-SCC-11B-CLASP1 (17)
6. UM-SCC-11B-CLASP1 (18)
7. UM-SCC-11B-LIG4 (4)
8. UM-SCC-11B-RNF8 (13)

Western blot analysis shows the expression of *RNF8* in the parental cell line, the Cas9 expressing cell line and the different knock-out cell lines.

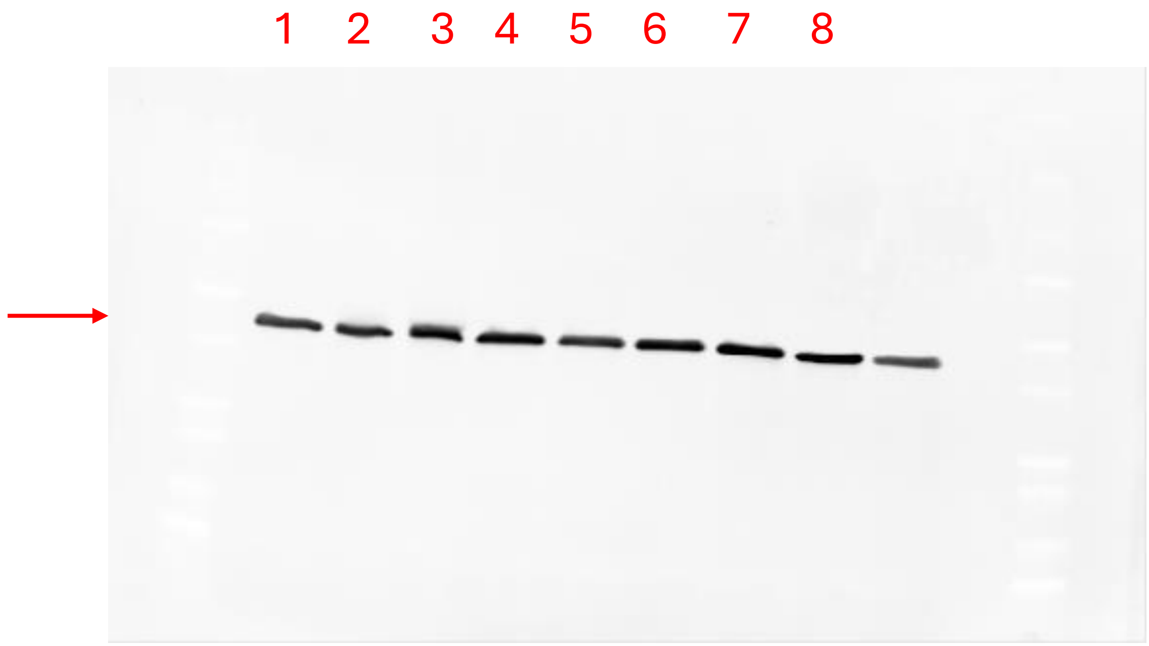

1. UM-SCC-11B-WT
2. UM-SCC-11B-Cas9
3. UM-SCC-11B-PRKDC
4. UM-SCC-11B-CLASP1 (12)
5. UM-SCC-11B-CLASP1 (17)
6. UM-SCC-11B-CLASP1 (18)
7. UM-SCC-11B-LIG4 (4)
8. UM-SCC-11B-RNF8 (13)

Western blot analysis shows the expression of *β-Actin* in the parental cell line, the Cas9 expressing cell line and the different knock-out cell lines.
